# The 26S proteasome is a multifaceted target for anti-cancer therapies

**DOI:** 10.18632/oncotarget.4619

**Published:** 2015-07-20

**Authors:** Tatyana A Grigoreva, Vyacheslav G. Tribulovich, Alexander V. Garabadzhiu, Gerry Melino, Nickolai A. Barlev

**Affiliations:** ^1^ St. Petersburg State Technological Institute (Technical University), St. Petersburng, Russia; ^2^ University of Rome Tor Vergata, Roma, Italy; ^3^ Institute of Cytology RAS, St. Petersburg, Russia

**Keywords:** proteasome, proteasome inhibitors, ubiquitin-dependent proteolysis, combined anti-cancer therapy

## Abstract

Proteasomes play a critical role in the fate of proteins that are involved in major cellular processes, including signal transduction, gene expression, cell cycle, replication, differentiation, immune response, cellular response to stress, etc. In contrast to non-specific degradation by lysosomes, proteasomes are highly selective and destroy only the proteins that are covalently labelled with small proteins, called ubiquitins. Importantly, many diseases, including neurodegenerative diseases and cancers, are intimately connected to the activity of proteasomes making them an important pharmacological target. Currently, the vast majority of inhibitors are aimed at blunting the proteolytic activities of proteasomes. However, recent achievements in solving structures of proteasomes at very high resolution provided opportunities to design new classes of small molecules that target other physiologically-important enzymatic activities of proteasomes, including the de-ubiquitinating one. This review attempts to catalog the information available to date about novel classes of proteasome inhibitors that may have important pharmacological ramifications.

## INTRODUCTION

### Importance of proteasomes as pharmacological targets

One of the hallmarks of tumor cells is the loss of cell cycle checkpoint control. In exchange for immortality, tumour cells lose the DNA replication fidelity and hence acquire an increased rate of genomic mutations. Consequently, transformed cells accumulate large quantities of misfolded or aberrantly overexpressed proteins, which may be toxic to cells. To cope with these problems, tumors cells enhance the expression of proteasomes to eliminate misfolded proteins. In this respect, it is, perhaps, not surprising, that several recent studies have found proteasome components associated with poly-ribosomes to ensure the tight quality control of newly synthesized proteins [1–3]. Since tumor cells are addicted to high levels of proteasomes, it was therefore prudent to test whether pharmacological inhibition of their proteolytic activities would affect the survival of tumors. Indeed, this approach proved successful for the treatment of aggressive hematopoetic tumors [4, 5]. However, these drugs do not perform well in solid cancers because of their high toxicity [6, 7]. This is due to the facts that, in general, higher doses of drugs are required to treat solid tumors. One of the plausible solutions to this is a combined treatment of malignancies with proteasome inhibitors and genotoxic or proteotoxic drugs. Rationally designed combinations of such drugs, in principle, should increase the efficacy of therapy and hence, decrease the dosage of individual drugs [8–10].

In this review we provide a snapshot of various aspects of proteasome functioning and biogenesis and discuss effects of small molecules that target proteasomes on different levels of their functioning. Finally, the effect of combined treatment with proteasome inhibitors and other anti-cancer drugs is discussed.

### Ubiquitin-dependent proteasome system

**Figure 1:**
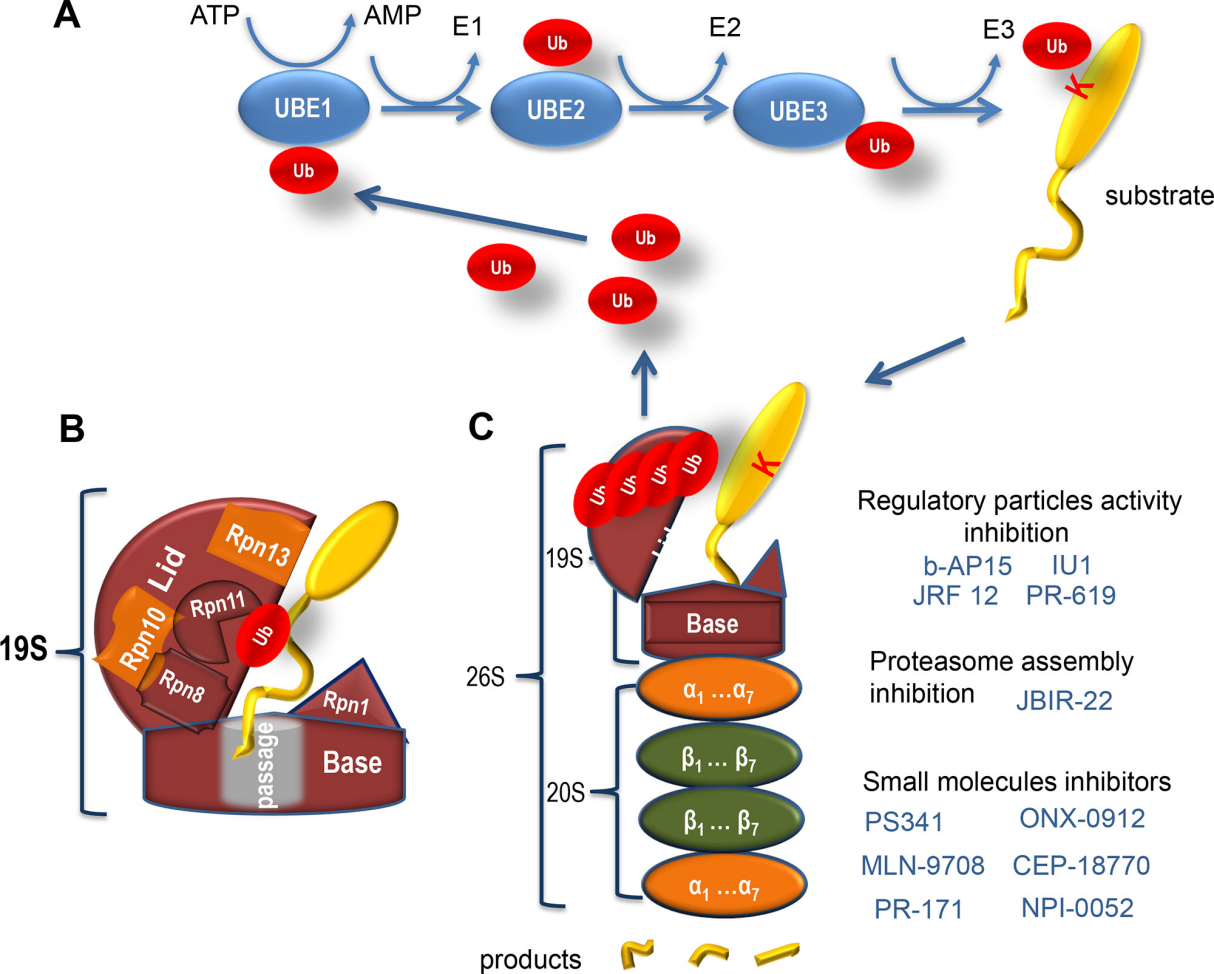
Ubiquitin-related protein degradation by proteasome **A.** Shown is a scheme of the Ubiquitin Proteasome System. In general, target proteins can be covalently modified on lysine residues with one or several small (76 amino acids) proteins, called ubiquitins (Ub) (shown in red). To be transferred onto the target lysine, Ub needs to be activated first by the Ubiquitin activating enzyme (E1) by forming a thio-ester bond with the latter. This reaction requires the energy of ATP. Subsequently, Ub is transferred to one of the Ubiquitin conjugating enzymes (E2), followed by an association with a substrate-specific Ubiquitin ligase (E3) enzyme, which covalently attaches Ub to the target protein. Importantly, Ubs can modify themselves thus forming poly-Ubs chains. The target protein should be labelled by a chain of at least four Ub (poly-Ub) to be efficiently recognized by the proteasome for its subsequent degradation. **B.** A schematic representation of the proteasomal 19S RP. Critical subunits of the base and the lid are indicated. Rpn10 and Rnp13 ubiquitin receptor subunits are shown in orange. A substrate protein (yellow) with the ubiquitin moiety (red) is also shown. **C.** Shown is the schematic structure of the proteasome and small molecules (blue) that affect its different activities and the assembly. The 19S RP is shown in brown, the 20S CP comprised of alpha- and beta-type subunits (orange and green, respectively) is also presented. A substrate protein (yellow) modified with ubiquitins (red) is depicted as well as its products of degradation (yellow fragments).

The ubiquitin proteasome system (UPS) provides a tight control of the intracellular protein degradation and turnover. A simplified scheme of the UPS action is shown in Figure 1. The mammalian ubiquitination system comprises several hundred different enzymes, including one E1, ~ 50 E2 and ~ 500 E3 ligases. The latter carry out specific ubiquitination of their target proteins to direct them to proteasomes, which are considered as cellular factories for protein degradation. Ubiquitin ligases as well as proteasomes localize both in the nucleus and cytoplasm of eukaryotic cells thus allowing them regulate the fate of a wide variety of different proteins [11]. Their intracellular distribution depends on the type of cells and tissues [12]. The functional versatility of proteasomes is defined not only by their dispersed cellular localization and high abundance (up to 1% of the total protein), but also by a number of enzymatic activities associated with them [13, 14]. Besides the proteolytic activitiy, which probably is the most studied one, proteasomes also possess with ATPase activity important both for unfolding the target protein and chromatin remodeling, RNAse activity, which might help to control splicing and mRNA expression levels, and de-ubiquitinase activity, required for stripping off the target protein from ubiquitins for its subsequent degradation [15–18].

### Structural organization of proteasomes

Versatility of proteasome activities is realized by its complex multisubunit structure (Figure 1B, 1C). The proteasome, also known as 26S proteasome based on its Svedberg sedimentation coefficient, is an ATP-dependent proteolytic complex with approximate molecular weight of 2.4 MDa. The proteasome consists of a cylindrical 20S complex (core particle / CP) and one or two regulatory 19S complexes (regulatory particle/RP) [12, 19] (see below).

The 20S CP is not able to hydrolyze proteins on its own, but cleaves small peptides and some unfolded proteins [19]. The 20S complex is shaped as a hollow cylinder formed by a stack of four rings, each of these rings consisting of seven different subunits. The two outer rings are made of the α-type subunits (α1-α7), which mediate the interaction with the 19S regulatory complex (Figure 1B). Besides this structural role, α-type subunits also exhibit endoribonuclease activity [20] and interact with several ubiquitin ligases and hydrolases [21]. The latter observations challenge the idea of α-type subunits carrying out only the structural role within the proteasome. The two inner rings are composed of the β-type subunits [22]. Subunits β5, β2 and β1 possess different proteolytic activities—chymotrypsin-, trypsin- and caspase-like, respectively. The chymotrypsin-like (CT-L) activity usually is the strongest one in proteasomes and cleaves peptide bonds after large hydrophobic amino acid residues. The trypsin-like activity (TL) provides cleavage after basic residues, and the peptidyl-glutamyl peptide-hydrolyzing or caspase-like (PGPH or C-L) activity - after acidic residues [23–25]. Furthermore, the combination of these two activities allows the proteasome to cleave peptide bonds both after branched and small neutral amino acids [23, 26]. Despite being evolutionary conserved the substrate specificity of β subunits may vary. In response to exposure of hematopoietic cells to pro-inflammatory signals such as cytokines, interferon gamma and others β subunits are substituted by their isoforms (β1i, β2i, and β5i). Such proteasomes are known as immunoproteasomes and their substrate specificity is altered relative to normal proteasomes [27, 28]. Thus, the unique collection of proteolytic activities provides the proteasome with versatility of destructing most of the peptide bonds available in nature.

The major role of the 19S complex is to prepare the substrate protein for degradation in CP (Figure 1B). Thus, 19S subunits exert the following functions: bind the ubiquitinylated substrate; remove the ubiquitin moieties from the protein; unwind the protein and direct it to the CP for subsequent proteolytic degradation [12, 19]. Biochemical studies dissected the RP into two subcomplexes, the ‘base’ and the ‘lid’ [22]. The center of the “base” is organized by six ATPase subunits Rpt1–6 (regulatory particle triple A 1–6), which directly contact the CP. They assemble into a heterohexameric ring formed by three pair of dimers: Rpt1/Rpt2, Rpt6/Rpt3, and Rpt4/Rpt5. The first two heterodimers are bound at their tips by two largest subunits of RP, Rpn1 and 2 (regulatory particle non-ATPase 1 and 2), respectively. Rpn1 provides a docking site for UBL (ubiquitin-like) domain- and UBA (ubiquitin-associated) - containing proteins such as Rad23 (radiation sensitive 23) that interact with various ubiquitinated proteins. Rpn2, in turn, is bound by another ubiquitin receptor, Rpn13, thus completing the “base” [11]. Collectively, the functional role of the base subunits is to recruit ubiquitinated proteins to the proteasome and mediate their local denaturation to facilitate the delivery to the CP. The “lid” complex consists of nine subunits Rpn3, and Rpn5–12. Current perception in the field is that the Rpn4 subunit, at least in yeast, does not stably associate with the 19S complex, but serves as a transcription factor orchestrating the expression of all proteasomal subunits. The main role of the “lid” complex is to de-ubiquitinate the target protein while translocaing it into the 20S complex [29]. Rpn9, Rpn5, Rpn6, Rpn7, Rpn3, and Rpn12 form a horseshoe-like structure with the heterodimer of Rpn8 and Rpn11 de-ubiquitinase placed in the vicinity of the AAA-ATPase hexamer. Such organization allows the substrate protein to be stripped from ubiquitin moieties immediately before entering into the 20S chamber. The second ubiquitin receptor, Rpn10, is positioned at the periphery of the lid, near subunits Rpn8 and Rpn9 [11]. In addition to Rpn11 ubiquitin hydrolase, which is also a metalloprotease, there are several other de-ubiquitinating proteins, USP14 (ubiquitin-specific protease 14) and UCH37 (ubiquitin C-terminal hydrolase 37), which loosely associate with the 19S regulatory particle yet modulate the trimming of ubiquitins [30].

### Additional protein complexes that regulate 20S CP

In addition to the 19S regulatory particle, there are several other protein complexes as well as individual proteins that activate the 20S particle by opening the passage to its catalytic chamber. These regulatory particles include PA28/11S regulator, COP9 and others [31–33].

The 11S regulatory particle is a ring-shaped homo- or hetero-heptamer protein complex consisting of the 28 kDa α, β and/or γ-type PA28 subunits. Similar to the 19S complex, the carboxyl termini of PA28 subunits induce conformational changes in the α-ring of 20S to open the gate [31]. Importantly, the 11S regulator does not contain any ATPases and therefore can promote the degradation of only short peptides but not the full-length proteins. The expression of the 11S particle is induced by gamma-interferon (γ-IFN) and often parallels the expression of the immunoproteasome β subunits. Together, they are responsible for the generation of peptides that bind to the major histocompatibility complex [34, 35].

Anothert regulatory particle, signalosome COP9/CSN (constitutive photomorphogenesis 9 signalosome) is a 430 kDa complex formed by eight subunits (CSN1–8). The eight subunits of the CSN share sequence homologies with the Rpn9, Rpn5, Rpn6, Rpn7, Rpn3, and Rpn12 polypeptides of the 26S proteasome “lid” complex. The CSN consists of six subunits with PCI (proteasome, COP9 signalosome, initiation factor 3) domains and two subunits with MPN (Mpr-Pad1-N-terminal) domains. COP9 interacts with cullin-containing E3 ubiquitin ligases and is necessary for their proper functioning. Also, it can bind protein kinases and deubiquitinating enzymes to regulate their degradation [32, 36].

Recently, a hybrid regulatory complex was found in mammals, which comprises the p97-related double-ring AAA+ ATPase Cdc48/p97/VAT and COP9. Interestingly, COP9 serves as a “base” and p97 as a “lid” of the complex, reminiscent of the structure of the 19S RP. This complex has been implicated in regulation of E3 ubiquitin ligases RNF8 and RNF168 [37] and exerts pleiotropic biological functions [38].

Not only complex protein structures modulate the activity of 20S proteasomes, but also individual proteins can induce conformational changes in the 20S CP rendering it active. For example, PA200, a 200 kDa protein of asymmetrical dome shape, binds to one or both ends of the 20S CP. It activates hydrolysis of short peptides, but not of the folded full-length proteins. However, the exact mechanism of PA200-mediated activation of the 20S CP is still under investigation. The yeast ortholog of PA200, Blm10 (former Blm3) was shown to be activated in response to DNA Damage (DD) and the deletion of Blm10 caused sensitivity to bleomycin, a DD-inducing drug. Taken together, these facts strongly suggest that the PA200 protein participates in DNA Damage Response (DDR), possibly engaging the 20S complex to remove damaged proteins [39]. In addition, PA200 was reported to mediate maturation of nuclear proteasomes, since knockout of Blm10 increased the intracellular level of half-proteasomes [11]. Collectively, the activity and specificity of the proteasome, besides the 19S RP, may also be regulated by protein modulators whose availability, in turn, is controlled in a cell type- and stimulus-specific manner.

### Proteasome assembly

Another level of regulation of proteasomal activity resides in the mechanisms of its assembly. Assembly of the 26S proteasome is a carefully regulated complex process. It starts with the formation of a seven-member α-ring, which serves as a template for the subsequent addition of precursor β-subunits to form a “semi-proteasome”. Maturation of the β-subunits concomitant with dimerization of the two half-proteasomes leads to generation of the 20S particle, which serves as a platform for further adjoining of the regulatory particles and the formation of the biologically active 26S proteasome. The efficacy and accuracy of each step of the proteasome assembly are regulated by specific chaperones. The assembly of the outer ring of the 20S CP is nucleated around the α5 subunit bound by the PAC3 homodimer (proteasome assembly chaperone). Because the latter binds to the inner part of the α-ring, it sterically hinders the addition of pro-β-subunits. Another pair of chaperones, PAC1/2, bind to the α-type subunits and prevents the premature dimerization of α-rings, which may interfere with proteasome biogenesis [40]. Upon the formation of the full-sized α-ring PAC3 and interacting with it PAC4 chaperones dissociate from the pro-proteasome [41]. The assembly of the β-ring is facilitated by POMP (proteasome maturation protein), which prevents the premature dimerization of precursors containing incomplete sets of β-subunits [42]. On completion of the β-ring, POMP is degraded and the two parts of half-proteasomes dimerize to form an enzymatically competent 20S CP [for review: 43 and references therein]. Another chaperone, Ecm29, a conserved HEAT-like repeat protein, controls the integrity of RP-CP assemblies [44]. Ecm29 serves as a scaffold protein that helps to remodel stalled RP-CP particles into regular enzymes. Following the completion of CP maturation, Ecm29 is degraded [43].

Thus, the speed and quality of the proteasome assembly is tightly regulated by chaperones, which may represent promising pharmacological targets.

## MODULATION OF PROTEASOME ACTIVITY

**Figure 2:**
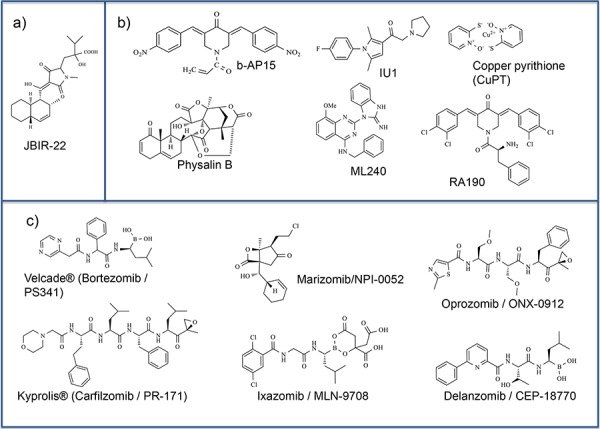
Proteasome small molecule modulators Chemical structures of various inhibitors of proteasomes. **a.** Inhibitor of PAC3 dimerization. **b.** Inhibitors of the 19S Regulatory particle. **c.** Proteasomal inhibitors studied in clinical trials.

As mentioned earlier, direct inactivation of 20S peptidase activities by blocking beta-subunits with pharmacological compounds often causes resistance due to mutations in the corresponding subunits [45]. Therefore, an alternative promising approach is to attenuate the proteasome activity by interfering with the assembly of the 20S and/or 26S particles. The PAC3 chaperone is essential for the proper formation of the 20S CP α-ring. To become physiologically active PAC3 requires homodimerization. Accordingly, blocking of the PAC3 homodimerization should result in attenuation of the CP assembly. Such small molecule inhibitor specific for PAC3 dimerization was described in [46]. In this study, the authors employed high-throughput screening (HTS) using an *in vitro* protein fragment complementation assay (PCA). This approach allowed them to identify an inhibitor of PAC3 dimerization - JBIR-22 (Figure 2A). The inhibitor was specific *in vitro* and showed cytotoxic effect on HeLa cells (human cervical carcinoma) with an IC50 value of 68 μM. Interestingly, this compound was active only when used for a long period of time (120 hrs), but showed no effect during the shorter treatment (48 hrs), suggesting that the assembly of 20S CP is a slow process [46].

### Modulation of activities specific for regulatory particles

One of the major functions of the 19S regulatory particle (primarily via the POH1/Rpn11 activity) is the removal of ubiquitins from the target protein before it enters 20S CP. If de-ubiquitination is blocked then the protein will not be degraded, because the narrow gateway to the catalytic chamber does not allow the passage of ubiquitinated peptides [45]. Thus, pharmacological inhibition of de-ubiquitinases seems to be another promising direction to interfere with the activity of proteasomes. Deubiquitinating enzymes (DUBs) play an important role in many processes, including DNA Damage response, DNA repair and transcription [47, 48]. They are also involved in several diseases, such as a hereditary cancer and neurodegenerations [49].

In general, DUBs can be divided into two classes according to their structures: metalloproteases and cysteine proteases. All metalloproteases, including POH1, contain the Ub-protease domain called JAMM (JAB1/MPN/Mov34 metalloenzyme) [50].

Cysteine proteases are divided into four families: ubiquitin-specific protease (USP), ubiquitin C-terminal hydrolase (UCH), Otubain protease (OTU), and Machado-Joseph disease protease (MJD) [50]. The differences in structure between representatives of these two classes reflect their specificity towards the linkage of poly-ubiquitins. Thus, the USP familily members (USP14) specifically recognize the free carboxyl-terminal double glycine motif of ubiquitin [51]. Accordingly, USP14 trims off only single ubiquitins, but is not able to remove the whole chain of ubiquitins from the substrate protein. On the other hand, UCH37, member of the UCH family, exhibits preferential specificity for the di-ubiquitin substrates. Conversly, POH1 can cleave polyubiquitin chains *en bloc* from unfolded proteasomal substrates [52].

Importantly, whereas knockdown of POH1 interferes with the proteasome assembly, depletion of either USP14 or UCH37 alone does not affect or even slightly enhances protein degradation rates. This suggests that the POH1 enzyme is critical for de-ubiquitination of the majority of substrate proteins by the proteasome, and USP14 and UCH37 play more specialized roles. Yet, the combined depletion of USP14 and UCH37 also inhibits proteasomal activity, which makes them attractive candidates for pharmacological intervention [53]. Although no small molecule inhibitor specific for POH1 has been discovered yet, a novel selective inhibitor of UCHL5 and USP14, b-AP15, has been developed recently (Figure 2B). b-AP15 was initially found in a course of cellular screening for small molecules that induce lysosomal apoptosis independently of the tumor supressor p53 [54]. This compound caused severe inhibition of the DUB activity (IC50 = 2.1 μM against Ub-AMC substrate [55]) without affecting the proteolytic activities of 20S CP [56]. b-AP15 treatment has been shown to slow down tumor progression in four various solid-state models *in vivo* [56]. Furthermore, b-AP15 inhibited proliferation of multiple myeloma (MM) cells resistant to bortezomib and synergized with suberoylanilide hydroxamic acid, lenalidomide, or dexamethasone [57].

Another compound, copper pyrithione (CuPT) (Figure 2B), was reported to target both 19S proteasome-specific DUBs, UCH37 and USP14, as well as 20S proteolytic peptidases. CuPT inhibited tumor growth *in vivo* and induced cytotoxicity *in vitro* and *ex vivo* [58]. Collectively, these published results strongly suggest that UCH37 and USP14 are very promising pharmacological targets.

That individual suppression of either UCH37 or USP14 leads to increased degradation rates makes specific inhibitors of these enzymes valuable for treatment of certain neurological conditions where the proteasome activity is severely attenuated. In agreement with this, a highly selective inhibitor of USP14, IU1, (Figure 2B) (IC50 of Ub-AMC hydrolysis by proteasome-bound USP14 is 4.7 ± 0.7 μM), enhanced the destruction of several important proteasome substrates (Tau, TDP-43) involved in the development of neurodegenerative diseases [59]. IU1 failed to significantly inhibit eight DUBs of human origin as well as Ub-AMC hydrolysis by proteasomes lacking USP14, which is attributable to UCH37. In the absence of proteasomes, USP14 was insensitive to IU1, indicating that IU1 binds specifically to the activated form of USP14 [59].

Several HTSs of small molecule libraries have also identified other compounds (HRF-3, piperlongumine and physalin B (Figure 2B)) that inhibit the UPS at levels other than the proteasome, probably affecting endoplasmic reticulum (ER)-associated protein degradation (ERAD) [60]. However, this interesting aspect is beyond the scope of this review.

Following the approach of indirect targeting of proteasomes, it can be assumed that blocking the ubiquitin-binding subunits within the 19S RP should also yield a significant malfunctioning of the proteasome. Indeed, recent report that bis-benzylidine piperidone RA190 (Figure 2B) covalently binds the ubiquitin receptor RPN13 in the 19S regulatory particle and inhibits the proteasome function, confirms the validity of this approach. Importantly, treatment of bortezomib-resistant MM cells with RA190 triggered rapid accumulation of polyubiquitinated proteins and cell death via endoplasmic reticulum stress-related apoptosis [61].

By the same token, targeting of the Rpt/ATPase-containing “base” of the 19S complex should also represent an attractive pharmacological approach. In line with this hypothesis is the fact that several low-molecular inhibitors of the p97/VCP/Cdc48 subunit of the Cdc48/COP9 regulatory complex have been identified to date [62, 63]. Noteworthy, mammalian AAA+ ATPase p97 (Valosin-containing protein (VCP), Cdc48 at yeast) is also capable of binding ubiquitin [3]. By means of HTS Chou with colleagues revealed a selective reversible ATP-competitive p97 inhibitor - N2, N4-dibenzylquinazoline-2, 4-diamine / JRF 12/ DbeQ with IC50 ranging from 1 to 7 μM depending on the cell type. Importantly, this compound induces apoptosis of cancer cells faster than the inhibitors of 20S CP proteolytic activites (e.g. MG132). Further analyses using structure–activity relationship (SAR) led to the identification of low molecular weight inhibitors ML240 (Figure 2B) and ML241, which selectively block degradation of p97-dependent proteasome substrates with IC50 = 900 nM and 3500 nM, respectively [62, 63].

Taken these results together, one can predict that targeting protein-protein intereactions of Rpn1 and Rpn2 with small molecules should also affect the activity of proteasomes.

### Small molecule inhibitors of proteasomal proteolytic activities

Since regulated proteolysis via proteasomes plays a key role in a number of pathological processes such as rheumatoid arthritis, cardiovascular diseases, bacterial and viral infections, cancer, and Alzheimer's disease, the field of proteasome inhibitors research has bloomed over the last decade [6, 64]. A list of various proteasome inhibitors currently used in biomedicine is shown in Table 1.

**Table 1:** Proteasome inhibitors

Compound/Origin (if natural)	Activity, IC50, nM			Type of assay	Ref.
	CT-L	T-L	C-L		
Peptide aldehydes					
Felutamide B (*Penicillium fellutanum*)	9.4	2000	1200	20S	[65]
TP-110	27			20S	[66]
MG132	68	4500	1400	20S	[66]
Tyropeptin A (*Kitasabospora sp*.)	40			20S	[65]
	1400	5000	68 000	20S	[66]
	Dose-dependent			PC12 cells	[67]
Syrbactins					
Glidobactin A (*Burkholderiales*)	15	>15		20S	[68]
Syringolin A (*Pseudomona syringae*)	1300	>1300	>1300	20S	[68]
Epoxyketones					
Epoxomicin (*Actinomycetes*)	5.7			20S	[65]
NC-022		300		20S	[69]
NC-001			500	MM1.R, NCI-H929	[70]
YU-102			+	20S	[71]
Vinyl esters					
HMB-LLL-VE	41	4210	>10 000	20S	[72]
Peptide Vinyl Sulfones					
MB1		+		20S	[73]
Natural polyphenols					
Epigallocatechin gallate / EGCG (tea polyphenols)	86–194			20S	[74]
	1000–10000			Living Jurkat T cells	[75]
Curcumin / diferuloylmethane (*Curcuma longa*)	1850	6230	3680	20S	[76]
	20000			HCT-116 and SW480 cells	[76]
Natural cationic porphyrins					
H2T4	750	530	460	20S and 26S	[77]
β-lactones					
Omuralide / clasto-lactacystin-β-lactone (*Salinospora tropica*)	29	690	8300	20S	[78]
	600			Living Jurkat T cells	[75]
Hydronaphthoquinones					
PI-083	1000	4500	4500	20S	[79]
	> 1000	> 4500	> 4500	Nude mice	[79]
Isothiocyanates					
BITC	4700	~10000	~10000	A549 cells	[80]
Triterpenoids					
Pristimerin (*Celastrus Maytenus*)	<100			20S	[81]
	50 000			H929 and U266 U266 cells	[81]
Celastrol (*Tripterygium Wilfordi, Celastrus Regelii*)	2500			A6 Xenopus cells, mammalian cells, human prostate cancer cells	[82]
	1000–5000			Nude mice PC-3 or LNCaP cells	[83]
Chalcones					
AM114	1000			20S	[84]
	1490			HCT116 p53 +/+ cells	[84]
Imidazoline derivatives					
TCH-013	2800		1600	20S	[85]
Carbamides and carbamates					
Hydroxyurea	1000			20S	[86]
DSF / disulfiram	2000			MDA-MB-231 cells	[87]
DSF Cu (II)	7500			20S	[88]
	>7500			26S	[88]
DSF Cd	3500			20S	[88]
	3200			hMCF 10 DCIS cells and PC-3 cells	[87]
Inorganics					
CuCl_2_	5100			20S	[89]

A summary of proteasome inhibitors known to-date grouped by: their origin (synthetic or natural), targeting specificity and IC50 against the proteasomal enzymatic activities (CT-L stands for ChymoTrypsin-Like, T-L stands for Trypsin-Like and C-L stands for Caspase-Like activity), and type of assay used for measuring the inhibitory effect. The compounds are also divided into classes according to their chemical structures (aldehydes, syrbactins, epoxyketones, etc.).

The pioneering first-in class proteasome-specific drug approved by FDA was Bortezomib / PS341 / Velcade^®^ (Figure 2C). It represents a dipeptidyl boronic acid-based compound that reversibly inhibits the chymotrypsin-like activity and to a lesser extent caspase-like activity of the 20S proteasome [90, 91]. Mechanistically, the boronic acid moiety of bortezomib forms a (pseudo)covalent bond with the nucleophilic hydroxyl side chain of Thr1 in the S1 pocket of the β5 subunit [92]. This dipeptidyl boronate has an IC50_CT-L_ = 2.4–8.2 nM for the 20S CP and 3.3 nM for 20S associated with the PA28 complex [93–96]. The drug was approved for the treatment of newly diagnosed multiple myeloma, relapsed/refractory multiple myeloma, and mantle cell lymphoma. The spectrum of anti-tumor activity of bortezomib is quite diverse and includes: upregulation of proapoptotic proteins (e.g., Noxa, IκB), inhibition of NFκB and its anti-apoptotic target genes, suppression of several anti-apoptotic proteins (e.g., Bcl-XL, Bcl-2, and STAT-3), down-regulation of expression of several proteins involved in DNA repair pathways, and induction of endoplasmic reticulum (ER) stress and pro-apoptotic Unfolded Protein Response (UPR). Bortezomib has potent chemo-/radio-sensitizing effects and can overcome traditional drug resistance in tumors when used in combination with potential chemotherapies. Although bortezomib showed great success in treating hematological malignancies, some patients relapsed after a positive initial response. Furthermore, bortezomib was found associated with several cytotoxicities (e.g. peripheral neuropathy) [97].

These observations have encouraged researchers to search for the next generation proteasome inhibitors that could overcome bortezomib resistance and have improved properties, reduced toxicities, and broader anticancer activities. The next, much improved, derivative of bortezomib was Carfilzomib / PR-171 / Kyprolis^®^ (Figure 2C). It was approved in 2012 by FDA for the treatment of relapsed and refractory multiple myeloma. Structurally, carfilzomib is a derivative of epoxyketone, which forms irreversible covalent bonds with the β5 subunit thus inhibiting the chymotrypsin-like activity (IC50_CT-L_ = 6.0 nM for 20S) [94, 96].

In addition to carfilzomib, several other second generation proteasome inhibitors have been developed and are now being actively tested in clinical studies: marizomib (salinosporamide A) [98], CEP-18770, MLN-9708 [99, 100] and ONX-0912 [101] (Figure 2C and Table 2). Marizomib is a natural product derivative that resembles lactacystin. In contrast to bortezomib and carfilzomib, marizomib irreversibly binds all three enzymatically active subunits of the proteasome (β1, β2, and β5), thus providing a durable and strong inhibition of both chymotrypsin-like (IC50_CT-L_ = 1.3 − 3.5 nM) and trypsin-like (IC50_T-L_ = 2.0 – 28 nM) activities [102–105]. Of note, marizomib was able to overcome bortezomib resistance in multiple myeloma and chronic lymphocytic leukemia cell models [106]. This important clinical property of marizomib could be due to its increased specificity toward caspase 8-mediated apoptosis compared to bortezomib [107].

**Table 2:** Completed clinical trials with proteasome inhibitors and other drugs

Compound	Sponsor	Combination	Condition	Phase	ClinicalTrials.gov Identifier
Marizomib/Salinosporamide A/NPI-0052	Triphase Research and Development I Corporation	Vorinostat	Non-small cell lung cancer, pancreatic cancer, melanoma or lymphoma	Phase 1, completed	NCT00667082
		-	Advanced malignancies	Phase 1, completed	NCT00629473
		-	Advanced solid tumor Malignancies or refractory lymphoma	Phase 1, completed	NCT00396864
Oprozomib/ONX0912	Onyx Therapeutics, Inc.	-	Advanced refractory or recurrent solid tumors	Phase 1, completed	NCT01129349
Ixazomib/MLN-9708	Millennium Pharmaceuticals, Inc.	-	Advanced nonhematologic malignancies	Phase 1, completed	NCT00830869
		-	Relapsed and/or refractory multiple myeloma	Phase 1, completed	NCT00932698
		-	Relapsed and refractory multiple myeloma	Phase 1, completed	NCT00963820
		Lenalidomide Dexamethasone	Newly diagnosed multiple myeloma	Phase 1 Phase 2, completed	NCT01217957
Delanzomib/CEP-18770	Cephalon	-	Solid tumours or non-hodgkin's lymphomas	Phase 1, completed	NCT00572637
		Lenalidomide Dexamethasone	Relapsed or refractory multiple myeloma	Phase 1 Phase 2, terminated	NCT01348919
		-	Relapsed multiple myeloma refractory to the most recent therapy	Phase 1 Phase 2, terminated	NCT01023880

Shown are the completed trials of anti-cancer therapies with proteasome inhibitors in combination with other drugs sponsored by various companies. Cancer conditions and accomplished phases are indicated.

Besides bortezomib and carfilzomib there are two other reversible peptide boronates proteasome inhibitors, delanzomib (CEP-18770) and Ixazomib (MLN-9708) [99, 100]. CEP-18770 (Figure 2C) has been investigated in Phase I clinical trials for the treatment of recurrent, advanced stage solid tumors, lymphoblastic leukemia and non-Hodgkin's lymphoma. It primarily inhibits chymotrypsin-like activity with IC50_CT-L_ = 3.4 nM [93]. Ixazomib is also currently being investigated in Phase I clinical trials for recurrent MM [100, 108]. Furthermore, oprozomib (ONX-0912) (Figure 2C) is another peptide epoxyketone proteasome inhibitor, which irreversibly inhibits chymotrypsin-like activity of proteasomes with IC50_CT-L_ ~10 nM and is currently in Phase I and II clinical trials for patients with solid tumors and hematological cancers [101]. Further clinical studies should define the anticancer efficacy of these second generation proteasome inhibitor drugs.

The immunoproteasome (20Si and 26Si) is a cytokine-inducible form of the proteasome in which β1, β2, and β5 subunits are replaced with the immunoproteasome-specific β1i, β2i, and β5i subunits, respectively [109]. It has been found that the levels of immunoproteasome in MM cells vary depending on the current status of disease. In general, MM cells express increased levels of immunoproteasome. On the contrary, relapsed myeloma and bortezomib resistant cells display suppressed levels of the immunoproteasome and increased levels of the constitutive proteasome [110]. IPSI-001, an immunoproteasome-β1i subunit-specific inhibitor, has been found to preferably inhibit the immunoproteasome 20Si activity over the constitutive 20S proteasome activity. Treatment of cancer cells with the IPSI-001 inhibitor promoted apoptotis. Other IPSIs, PR-924 and PR-957 were able to overcome resistance to bortezomib in the preclinical setting, suggesting that they may provide an alternative approach to overcome the resistance to bortezomib [110, 111].

## TREATMENT OF TUMORS WITH PROTEASOME INHIBITORS COMBINED WITH OTHER DRUGS

It should be noticed that, in addition to the development of more effective and specific inhibitors of the proteasome, there is another productive strategy to increase the efficacy of anti-cancer therapy - via exploration of various combinations of proteasome inhibitors with other anti-cancer drugs. Below, we mention several most popular therapeutic combinations that involve proteasome inhibitors (Table 2).

### Proteasome inhibitors in combination with immunomodulators

Proteasome inhibitors are often combined with immunomodulators, another class of drugs that is actively used to control cancer, for example, lenalidomide, dexamethasone, and prednisone. In such studies the most commonly used proteasome inhibitors are bortezomib (Velcade^®^ and carfilzomib (Kyprolis^®^) that have already been approved for clinical use, but other combinations are also being tested. In particular, xenograft studies showed promising results for the treatment of MM with delanzomib combined with immunomodulators [112]. This scheme of treatment is also applicable not only to MM, but, for example, to prostate cancer [113]. An anti-cancer effect of proteasome inhibitors is significant even with the joined application of immunomodulators and alkylating agents (e.g. prednisone and melphalan) [114].

### Proteasome inhibitors in combination with HDAC inhibitors

Combined therapies based on the proteasome inhibitors are gaining their momentum in clinical oncology and have been tested in various hematopoetic tumors including non-Hodgkin's lymphoma [115], T-cell lymphoma [116], mantle cell lymphoma [117] and other types of cancer. Among the most popular partners in combination with proteasome inhibitors to treat the hematological malignancies are histone deacetylase (HDAC) inhibitors [118]. These inhibitors belong to a very promising class of epigenetic regulators and are being actively investigated in conjunction with many drugs, including not only bortezomib, but also other proteasome inhibitors, e.g. MG132 and epoxomicin [119]). The effect of combined therapy of proteasome and histone deacetylase inhibitors was also investigated on solid tumors such as prostate cancer [119], operable non-small lung carcinoma (NSCLC) [120], colorectal cancer [121], and hepatoma liver cancer [122].

### Proteasome inhibitors in combination with HSP-90 inhibitors

Clinical trials (phase I/II) have demonstrated the efficacy of combined application of bortezomib with the inhibitor of heat-shock protein 90 (HSP-90), 17-AAG [123]. This is first-in-class semi-synthetic analogue of the natural HSP-90 inhibitor, geldanamycin. Although 17-AAG performed poorly in clinical trials, it displayed strong inhibitory effect on the expression of IL-6R, IGF-IRβ and associated signaling molecules, including the pro-survival PI3K/AKT signaling pathway [124]. This suggests that the HSP90 inhibitors have a promising future in clinics as anti-cancer drugs, pending the reduction of their cytotoxicity and increased solubility. In this respect, KW-2478, a novel HSP90 inhibitor, is being currently investigated in pre-clinical trials. Interestingly, the combination of KW-2478 together with bortezomib showed higher efficacy in treatment of MM compared to the treatment with any of these drugs alone [125]. A similar effect was observed when bortezomib was used with an inhibitor of HSF1 (heat shock transcription factor 1), which transcriptionally controls the expression of HSP genes, including HSP90 [126].

### Proteasome inhibitors in combination with kinase inhibitors

It is well established that the phosphoinositide 3-kinase (PI3K)/AKT-mTOR signaling pathway plays an important role in the survival and tumor chemoresistance [127]. Accordingly, direct inhibition of the PI3K pathway in cancer cells causes anti-proliferative and cytotoxic effect, especially when combined with other drugs. In line with this notion is the observation that the anti-cancer efficacy of pan-PI3K inhibitor, SF1126, is augmented in MM cells when combined with bortezomib or MG132 [128, 129]. Likewise, the LY294002 PI3K inhibitor was shown to aid overcoming resistance to bortezomib, yet it did not demonstrate any apoptotic effect when used as monotherapy [130]. Finally, a multi-kinase inhibitor sorafenib, which acts similar to LY294002 on a wide range of different tumors, was shown to synergize with bortezomib by affecting Akt and JNK signaling pathways [131]. Collectively, it would be prudent to say that the combinatorial treatment of cancers with PI3K and proteasome inhibitors, although in its early stage of development, has very promising future.

### Proteasome inhibitors and ER stress

The anti-proliferative effect of many drugs is associated with the induction of endoplasmic reticulum (ER) stress, upon which misfolded proteins travel from the ER back to the cytosol for destruction by proteasomes in the ubiquitin-dependent manner. The anti-cancer effect of treatment with HSP-90 inhibitors together with bortezomib is based on their ability to synergistically induce ER stress. Surprisingly, ER stress can also be induced by HIV protease inhibitors, such as nelfinavir [132], or Calp Inh IV, and PD150606 [133]. The latter two blunt the activity of calpain and enhance the effects of proteasome inhibition [134].

It should be noted that resistance of MM cells to Bortezomib could be explained in part, by incomplete inactivation of the (ER)-associated protein degradation (ERAD) system and activation of non-proteasomal protein degradation pathways. In this respect, it is important to mention that the direct inducer of ER stress, eeyarestatin, enhances the cytotoxic effect of bortezomib on MM cells [135]. Furthermore, macrolide antibiotics, e.g. concanamycin A, erythromycin, clarithromycin and azithromycin, which do not exhibit cytotoxicity on their own, synergize with bortezomib [136]. The molecular mechanism behind this phenomenon utilizes the ability of these antibiotics to inhibit autophagy. Thus, inhibition of both autophagy and the proteasome activity by Bortezomib in MM cells results in complete ERAD inhibition and hence, increased cytotoxicity [133].

All these indicate that the use of combined therapy not only may significantly extend the range of diseases that are amenable to therapeutic action of proteasome inhibitors but also overcomes the problem of resistance in patients due to the simultaneous intervention of several cellular processes.

## CONCLUSIONS AND PERSPECTIVES

As cancer cells are more sensitive to proteasome inhibition than normal cells due to their elevated proliferation rates and the loss of translation quality control, the pharmacological targeting of proteasomal activities provides a new promising avenue for basic and clinical research. Supporting this notion is the fact that the clinical efficacy of bortezomib in treating hematopoetic malignancies has been well established. However, this preliminary success should be considered with caution. The majority of patients who initially responded to chemotherapy with bortezomib eventually became refractory to the drug, because of the increased rate of mutations in the target β5 subunit of the proteasome. This fact is a strong warning that the direct inhibition of the proteasome-mediated proteolytic activity may be too mutagenic to be considered as a long-term treatment of cancer. In this respect, it should be mentioned that the targeting of non-proteolytic proteasomal activities (de-ubiquitination and ATP remodeling) and even interfering with the proteasome assembly with small molecules may yield a better, more specific therapy, which will have less side effects. This direction has just only begun to actively develop.

Another issue of mono-therapy with bortezomib is its various efficacy depending on the tissue: although it is very potent in treating hematopoetic malignancies, its success in solid tumors has been rather limited. Whether the inert response to bortezomib is an intrinsic property of solid tumor cells, or this is due to the drug itself is currently unknown. Nevertheless, there is substantial evidence that the combination of proteasome inhibitors with conventional chemotherapeutic drugs help to overcome drug resistance of solid tumor cells [137]. Bortezomib was also shown to increase radiosensitivity of cancer cells, which makes the combination of bortezomib with radiotherapy or radiomimetic drugs a promising therapeutic tool [138]. Future studies will show the clinical relevance of bortezomib-based combinatorial therapies.

In sum, development of novel proteasome inhibitors with various specificities as well as novel drug combinations should help to address some of the key issues with bortezomib and offer possibilities for future anti-cancer therapies.
